# Surgical management of epilepsy due to cerebral cavernomas using neuronavigation and intraoperative MR imaging

**DOI:** 10.1179/016164113X13801151880551

**Published:** 2013-12

**Authors:** Bjoern Sommer, Burkhard Sebastian Kasper, Roland Coras, Ingmar Blumcke, Hajo Martinus Hamer, Michael Buchfelder, Karl Roessler1

**Affiliations:** 1 Department of Neurosurgery, University Hospital Erlangen, Erlangen, Germany; 2 Department of Neurology, Epilepsy Center, University Hospital Erlangen, Erlangen, Germany; 3 Department of Neuropathology, University Hospital Erlangen, Erlangen, Germany

**Keywords:** Cavernous hemangioma, Epilepsy outcome, Intraoperative high field MRI, Surgical morbidity

## Abstract

**Objectives:**

Cure from seizures due to cavernomas might be surgically achieved dependent on both, the complete removal of the cavernoma as well as its surrounding hemosiderin rim. High field intraoperative MRI imaging (iopMRI) and neuronavigation might play a crucial role to achieve both goals. We retrospectively investigated the long-term results and impact of intraoperative 1·5T MRI (iopMRI) and neuronavigation on the completeness of surgical removal of a cavernous malformation (CM) and its perilesional hemosiderin rim as well as reduction of surgical morbidity.

**Methods:**

26 patients (14 female, 12 male, mean age 39·1 years, range: 17–63 years) with CM related epilepsy were identified. Eighteen patients suffered from drug resistant epilepsy (69·2%). Mean duration of epilepsy was 11·9 years in subjects with drug resistant epilepsy (*n*  =  18) and 0·3 years in subjects presenting with first-time seizures (*n*  =  8). We performed 24 lesionectomies and two lesionectomies combined with extended temporal resections. Seven lesions were located extratemporally.

**Results:**

Complete CM removal was documented by postsurgical MRI in all patients. As direct consequence of iopMRI, refined surgery was necessary in 11·5% of patients to achieve complete cavernoma removal and in another 11·5% for complete resection of additional adjacent epileptogenic cortex. Removal of the hemosiderin rim was confirmed by iopMRI in 92% of patients. Two patients suffered from mild (7·7%) and one from moderate (3·8%) visual field deficits. Complete seizure control (Engel class 1A) was achieved in 80·8% of patients with a mean follow-up period of 47·7 months.

**Discussion:**

We report excellent long-term seizure control with minimal surgical morbidity after complete resection of CM using our multimodal approach.

## Introduction

Cavernomous malformations (CM) of the brain are vascular malformations with an estimated prevalence between 0·4 and 0·9%,^1^ appearing mainly as singular supratentorial lesions.^2^ Their biology is usually benign without changes in size, although the potential for growth and recurrent bleeding is well documented.^3^^–^7 Importantly, 35–80% of all patients with supratentorial CMs are affected by first-time seizures, of which up to 40% are resistant to antiepileptic drug treatment.4,^8^^–^11 In patients with drug resistant epilepsy, complete lesionectomy with additional removal of the hemosiderin rim surrounding the CM has been discussed as a predictor of favourable postsurgical seizure control.^11^^–^13 As the five-year risk of epilepsy after first-ever seizures reaches 94% in patients with CM,^14^ early surgical resection was proposed as valuable alternative to medical treatment. However, surgery may be dangerous or ineffective in patients with CMs located in eloquent brain areas or in CMs with broad hemosiderosis. In these patients, intraoperative MRI (iopMRI) and functional neuronavigation (FN) may be helpful to achieve successful surgical treatment.^15^^–^17 Resections adjacent to eloquent brain regions can be performed more safely by applying FN and incomplete resection can be detected by iopMRI.^18^ This concept was termed ‘multimodal navigation’ as reported earlier by our group.^19^ Thus, we compared our long-term results with this technique regarding the number of complete CM plus hemosiderin rim resections and postsurgical seizure control to existing series. Moreover, we expected lower rates of surgical morbidity in patients with eloquently located CMs.

## Patients and Methods

### Counselling policy for CMs

Our policy in counselling CM patients is to recommend surgery in cases with neurological symptoms, e.g. deficits due to overt hemorrhage or space occupying lesion or to treat patients with medically intractable epilepsy. Additionally, we offer surgery to patients with CMs after a single seizure as an alternative to medical treatment for seizure control because of the high chance of success of surgery compared to anti-epileptic drug (AED) treatment. Due to our technical equipment and past experience, we are also able to recommend surgery even in highly eloquent regions.15,^18^

### Study population

Between September 2002 and March 2012, we operated on 26 epilepsy patients with a single supratentorial CM. Drug resistant epilepsy was documented in 18 of 26 patients (69·2%) according to Kwan and colleagues.^20^ These 18 patients underwent extensive presurgical evaluation at the Epilepsy Center, University Hospital Erlangen including video-EEG monitoring, neuropsychological testing and high resolution 1·5–3·0 T MRI scanning.^21^ Intracarotid amobarbital test (Wada test) was performed in eight patients for assessment of language lateralization and memory performance. In five subjects, we applied functional MRI (fMRI) and diffusion tensor imaging (DTI) to localize eloquent brain areas and fiber tracts. In one patient, the epileptogenic focus determined by preoperative magnetoencephalography (MEG) was additionally embedded into neuronavigational data. The remaining eight patients were referred to our hospital after experiencing first-time sporadic seizures. They received routine scalp EEG and high resolution MR imaging. The results of these investigations were discussed at an interdisciplinary conference from which the surgical treatment plan was defined.

### Preoperative image acquisition

Preoperative magnetic resonance imaging (MRI) scans were obtained in every patient using a 1·5 or 3·0 Tesla MRI scanner (MAGNETOM Sonata Maestro Class and MAGNETOM Trio, Siemens Healthcare, Erlangen, Germany). In addition to standard MR sequences, FLAIR and gradient-echo T2 weighted images identified the lesions with the surrounding hemosiderin rim in all cases. Nineteen lesions were located in the temporal lobe and seven extratemporally.

### Functional data image fusion

**Figure 1:**
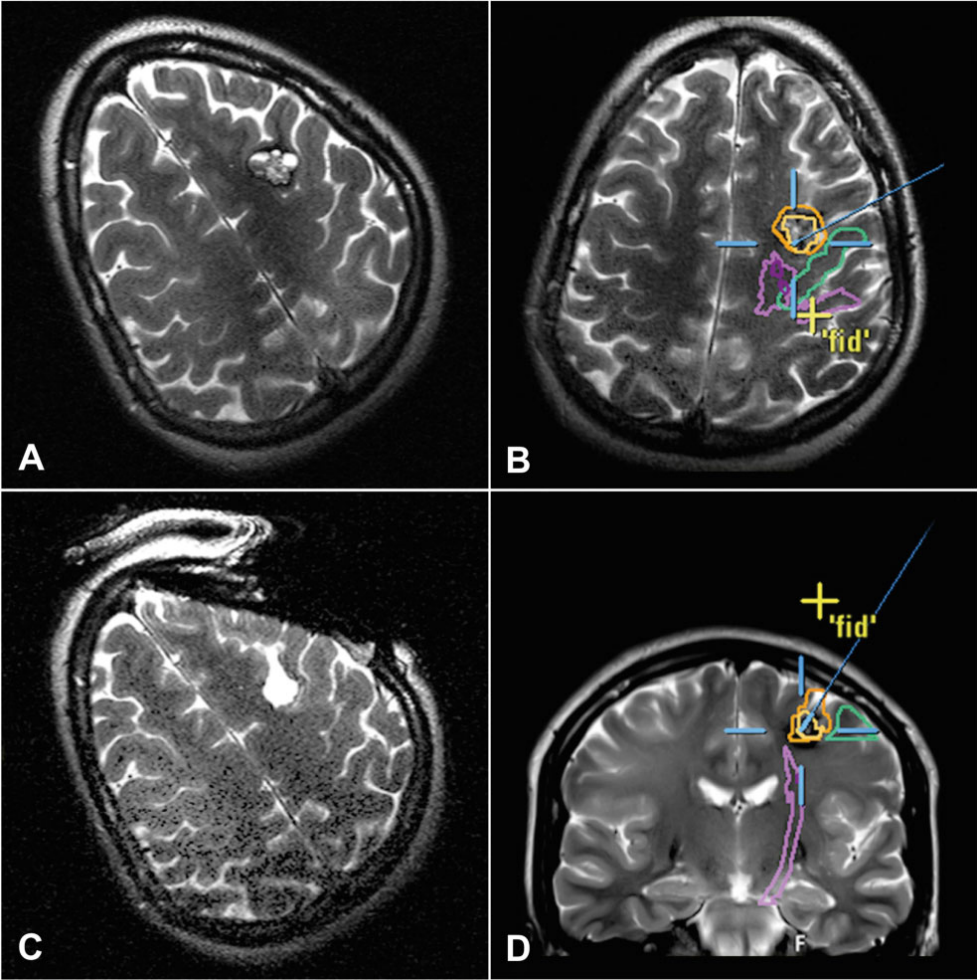
Axial (A–C) and coronal (D) T2-weighted intraoperative 1·5T MRI sequences of patient No. 18 with a precentral cavernous hemangioma. Prior to beginning of surgery (B, D), the cavernoma (orange) with the surrounding hemosiderin rim (ocher) has been segmented manually and implemented into the neuronavigation plan along with functional magnetic resonance imaging and diffusion tensor imaging data of the primary motor area (green) and the pyramidal tract (purple). The ideal trajectory and the target point (cross) is marked in light blue. Images A and C document complete extended lesionectomy [see online for colour version].

In five patients with lesions close to eloquent brain areas, we applied fMRI and MEG during the investigational period prior to surgery as described previously.13,15,^22^ Furthermore, DTI was used for reconstruction and visualization of the neuronal fiber tracts with the navigation planning software iPlan 2·6 (BrainLab, Feldkirchen, Germany). The procedure of localizing fiber bundles was demonstrated previously by our group.23,^24^ In two patients, the Wernicke language area, language and visual tracts were calculated; motor areas and/or pyramidal tracts were visualized in three more patients (Table 1, Fig. 1). For determination of the least distance between the calculated functional data and the segmented cavernoma including its hemosiderin rim, we screened the appropiate iopMRI slices in every axis and measured the distance manually using the ‘ruler’-function of the iPlan software.

**Table 1:** Patient and lesion characteristics in 26 patients with cavernous malformations

Patient No.	Sex	Age (years)	Time to OP (years, month)	Side	Lesion location	Lesion volume (cm^3^)	Thickness of hemosiderin fringe (mm)	Resection of hemosiderin fringe?	Functional data	Update of iopMRI navigation?	Operating time (minutes)	FU (months)	Drug resistant epilepsy?	Seizure outcome (Engel)
1	f	55	30, 0	R	temporal*	0·48	3·3	yes		yes	515	23	yes	3A
2	m	33	0, 1	R	temporal	0·34	3·4	yes		yes	392	116		1A
3	f	30	20, 4	L	temporal	6·73	3·6	partial			335	102	yes	1A
4	f	19	0, 7	R	temporal	2·0	1·6	partial			227	100		1A
5	f	59	2, 11	L	temporal	0·06	0·7	yes			215	100	yes	1A
6	m	32	2, 11	R	temporal	0·12	1·9	yes			260	74	yes	1A
7	m	63	2, 0	L	temporal	1·08	2·9	yes		yes^§^	150	69	yes	1A
8	m	24	0, 1	R	parietal	1·56	1·5	yes	M		145	68		1A
9	m	22	9, 0	R	temporal	0·27	5·0	yes			206	66	yes	1A
10	f	62	38, 0	R	temporal	2·31	1·6	yes			264	59	yes	2B
11	m	18	0, 9	R	parieto-occipital	45·51	1·0	yes	W,L,V		185	54		1A
12	f	21	3, 0	R	occpital	3·81	3·6	yes	W,L,V		120	54	yes	1A
13	f	48	15, 0	L	temporal	2·55	2·8	yes			264	52	yes	1A
14	m	41	0, 1	R	frontal	8·74	2·0	yes			214	12		1A
15	m	45	7, 0	L	temporal**	0·21	5·2	yes		yes^§^	216	37	yes	2B
16	f	39	15, 0	R	temporal	0·08	0·9	yes			127	35	yes	3A
17	f	35	0, 1	L	temporal	1·68	2·4	yes			146	27		1A
18	f	26	3, 11	R	frontal	2·29	1·7	yes	M,P		101	18	yes	1A
19	m	35	0, 3	L	frontal	0·13	1·0	yes	P		97	17		1A
20	f	17	1, 4	L	temporal	3·3	1·0	yes			220	14	yes	1A
21	m	49	1, 0	R	frontal	0·39	1·8	yes			175	13	yes	4C
22	f	51	0, 3	L	temporal	0·08	2·5	yes		yes	218	15		1A
23	f	50	13, 0	L	temporal	3·74	2·2	yes			245	11	yes	1A
24	f	38	30, 0	L	temporal	0·53	0·8	yes			111	10	yes	1A
25	m	46	13, 0	R	temporal	0·1	1·3	yes			198	52	yes	1A
26	m	59	7, 0	L	temporal+frontal**	0·44	2·2	yes		yes^§^	190	41	yes	1A

Lesion location: *dual pathology with additional hippocampal sclerosis, **lesionectomy of remnant cavernoma; Functional data: M  =  motor area, W  =  Wernicke’s area, L  =  language tract, V  =  visual tract, P  =  pyramidal tract; Update of iopMRI navigation: ^§^patients with additional resection of suspected perilesional epileptogenic brain tissue; FU  =  follow-up.

### MR imaging in the OR

All procedures were performed under general anesthesia. At the beginning of surgery, the patient’s head was fixed in a MRI-compatible ceramic head holder. Then, the patient was moved into the 1.5 Tesla iopMRI scanner (Magnetom Sonata Maestro Class, Siemens Healthcare, Erlangen, Germany) in order to acquire a current imaging data set. The preoperative scan sequences in the OR included a T1-weighted MPRAGE sequence (TE 4·38 ms, TR 2020 ms, matrix size of 128×128 (interpolated to 256×256), FOV 250 mm, slice thickness 1 mm, slab 16 cm), T2-weighted coronal and transversal images (TE 98 ms, TR 6520 ms, matrix size 512×307, FOV 250 mm, slice thickness 3 mm) and DTI sequences (TE 86 ms, TR 9200 ms, matrix size 128×128, FOV 240 mm, slice thickness 3 mm). Then, the patient was shifted back to the operating position outside the 5 Gauss line. When the surgeon had the impression of radical resection, the next intraoperative scans were performed with the head open and with the same protocol as the preoperative iopMRI sequences. The mean operating time was 213±93 minutes, while overall scan time for the acquisition of intraoperative MRI sequences (T1-weighted MPRAGE, T2-weighted coronal and transversal, DTI) was 14 minutes (Table 1).

### Intraoperative neuronavigation

**Figure 2:**
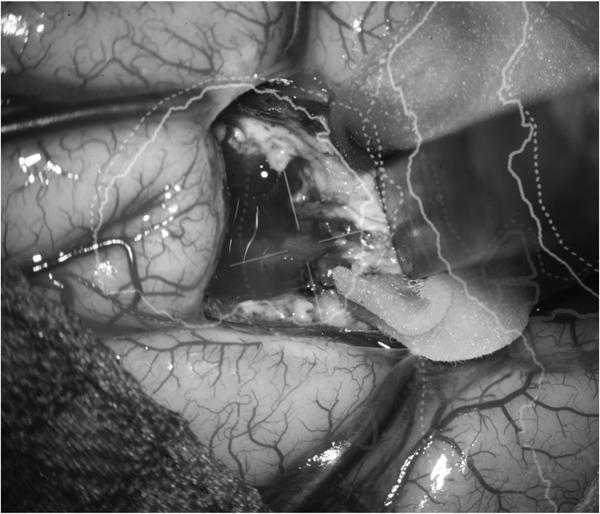
View through the operation microscope with displayed neuronavigational data. At this point of the operation, the outlined cavernoma within the focal plane of the microscope (light blue line) has been resected almost completely. The remaining characteristic mulberry appearance of the lesion can be seen just below the target point (light blue cross). Primary motor area is depicted in light green and pyramidal tract in purple.

After transmission of current MRI data from the 1·5 T scanner to the navigation software, preoperative functional MR images and DTI data were merged with the new scans. The lesion as well as the surrounding hypointense rim were manually segmented (Fig. 1) by using the neuronavigation software (iPlan 2·6, Brainlab AG, Feldkirchen, Germany). Afterwards, navigational data were transferred to an OPMI Pentero operation microscope (Zeiss, Oberkochen, Germany) and projected into the surgeon’s field of view as 3 D contours (Fig. 2). Boundaries of neuronavigation data were superimposed and drawn on the scalp for planning skin incision and craniotomy.

### Surgical technique

Microsurgical approaches were used for lesionectomies with the main goal of minimizing collateral brain tissue damage en route to the cavernous malformation (CM). With the aid of intraoperative neuronavigation, we checked if our predetermined surgical approach matched with the displayed boundaries of segmented pathological tissue on the scalp in the viewing field of the microscope. After we had the impression of complete resection, an intraoperative MRI scan was performed. In case of subtotal lesionectomy and/or incomplete removal of the hemosiderin rim, we performed an update of neuronavigation. A second intraoperative MRI scan confirmed complete resection before the closing procedure.

### Postoperative neurological evaluation and epilepsy outcome

Neurological deficits were assessed by immediate pre- and postoperative clinical investigation as well as 3 months later and recently for this report. We defined disabilities such as quadrantanopsia, minimal mnestic aphasia, or latent monoparesis as *mild neurological deficits*, a visual field loss of 25–50% or fluctuating aphasia as *moderate neurological deficits* whereas, for example, manifest hemiparesis or complete hemianopsia were considered as *severe neurological deficits*. For definition of epilepsy outcome, we used the most recent Engel classification.^25^ An *excellent outcome* was defined as Engel class Ia, whereas a *favourable outcome* included Engel’s classes I and II. The categories III and IV were assigned to the term *poor outcome*. Furthermore, we considered the number of AEDs before surgery and at the time of follow-up.

## Results

Data from 26 patients (14 female, 12 male) with CMs who had presented with sporadic or drug resistant seizures were analyzed (Table 1). Mean age at operation was 39·1±14·5 years. Presurgical evaluation revealed 18 patients (69·2%) with medically intractable epilepsy, of whom the mean epilepsy duration was 11·9±10·9 years. The combination of simple and complex partial seizures (5/26) or additional secondary generalized seizures (5/26) were the predominat seizure types (39%, 10/26) of our series (Table 2). The mean follow-up of our patient population was 47·7±32·2 months. Twelve resections were performed on the left hemisphere. Diagnosis of a CM was neuropathologically confirmed in all patients. One patient (No. 1) suffered from dual pathology with additional hippocampal sclerosis, where the resection of temporo-mesial structures was performed simultaneously to CM removal. In two of the patients who received incomplete resection in another hospital, we performed a modified temporal (No. 15) and fronto-temporal (No. 26) resection including perilesional epileptogenic cortex according to our presurgical epilepsy work-up.

**Table 2:** Summary of seizure and follow-up characteristics

Patients (*n* = 26)		
Mean age at surgery (years±SD)	39·1±14·5	

Mean duration of epilepsy (years±SD)		
*in subjects with drug resistant epilepsy (n = 18)*	11·9±10·9	
*in subjects presenting with first-time seizures (n = 8)*	0·3±0·3	

Number of AEDs taken *prior to surgery*	1·2±0·7	
*post surgery*	0·6±0·6	

Seizure type	*n* (%)	frequency per month
simple partial (sp)	1 (3·8)	4
complex partial (cp)	4 (15·4)	0·16–120
secondary generalized (sg)	4 (15·4)	0·16–1
sp+cp	5 (19·2)	1–80
sp+sg	4 (15·4)	0·16–5
cp+sg	3 (11·5)	0·5–90
sp+cp+sg	5 (19·2)	0·16–60

### Amount of resection

The mean lesion volume of the all patient’s CMs was 1·3±0·4 cm^3^ and the mean thickness of the hemosiderin-stained rim was 2·2±1·2 mm (Table 1). Out of 26 cases, an incomplete resection of the cavernoma or the hemosiderin rim was found in one (case No. 1) and two (No. 2 and 22) patients, respectively (3/26 or 11·5%), by means of the first iopMRI resection control scan. In three other patients (No. 7,15,26), the intended resection extent of perilesional epileptogenic cortex defined according to our presurgical work-up was not accomplished although removal of the entire cavernoma and its hemosiderin rim was achieved. Thus, an update of neuronavigation was performed in these six patients using images from the first iopMRI control scan (Table 1). After re-segmentation of residual lesion, total resection was achieved and confirmed by a second intraoperative MRI in all of those patients, raising the total lesionectomy rate to 100% of patients. Residual hemosiderin was left *in situ* in two patients (Table 1) due to the eloquent location of this tissue.

The registration of the neuronavigation system was accomplished with an accuracy of 1·4±0·9 mm and correlation of anatomical landmarks with navigation data confirmed correct image registration in all cases. In five patients with highly eloquent located CMs (No. 8,11,12,18,19), the minimal distance between eloquent brain areas and the segmented epileptogenic lesion was 8·6 mm mean.

### Seizure outcome

Twenty-one patients (80·8%) were completely seizure free (Engel class IA), 12 of those patients (46%) without antiepileptic medication at the time of last follow-up. *Favourable* seizure outcome was achieved in additional 2 patients (Table 1). In 11% of patients, we observed *poor* seizure control and one patient (No. 21) had aggravation of seizure frequency and intensity (Engel class IVC).

A subgroup analysis showed that 13/18 patients (72·2%) with preoperatively medically resistant epilepsy were seizure free compared to 8/8 patients (100%) who suffered from sporadic seizures preoperatively. Of the seven patients with extratemporal location of their CM, six patients (85·7%) had a complete seizure control (Engel IA).

Four-year follow-up data were available from ten patients, all of which had Engel class I or II seizure outcome (Table 1).

### Neurological and general medical complications

Three out of twenty-six patients suffered from visual field deficits, which were mild in two (7·7%) and moderate in one (3·8%) patient. Additionally, one patient experienced a soleal vein thrombosis and one an aseptic meningitis (7·7% general medical complications). No surgical revision was necessary or additional morbidity occurred.

## Discussion

This consecutive case study evaluated the application of iopMRI and FN during neurosurgical resection of epilepsy-related CM. A direct benefit of iopMRI was reported for 23% of patients, whose CMs would have been incompletely removed and thus would have had a lesser chance to become seizure free.8,^26^ FN proved its benefit indirectly, as all lesions were targeted and removed without major surgical morbidity, even when addressing eloquent cerebral areas. The combination of both advanced technologies improved long-term seizure outcome to 80·1% Engel class 1A at a mean follow-up time of almost 4 years.

### Treatment of CM associated with epilepsy syndromes

Although there is still much debate whether a conservative or operative strategy should be preferred in patients with CMs and new-onset seizures,3,^27^^–^29 early microsurgical resection is favoured in patients with drug resistant epilepsy, increased bleeding risk, and mass-effect with new neurological deficits.^2^^–^4,^8^–11,13,26,^28^–^30^ Importantly, the CM has to be removed completely, as emphasized in a recent meta-analysis of 1226 CM patients with lesion-related seizures.^8^ Here, gross-total resection was revealed as one of the major prognostic factors of seizure control. This underlines the usefulness of immediately verifying completeness of lesion removal by iopMRI.

### Intraoperative MRI and neuronavigation

In a previous study, we already proved the benefit of low field (0·2T) intraoperative MRI in patients with supratentorial cavernomas in combination with different neuronavigation systems.^15^ However, only 63% of those patients were seizure free after a mean follow-up of 10 months, partly due to 0·2T iopMRI being not capable to visualize hemosiderin as high field (1·5T) MRI does. At the time of installation of an intraoperative 1·5T high field MR suite at our clinic in 2002, we started to test the hypothesis that 1·5T iopMRI in combination with neuronavigation may extend the resection amount of CMs and lead to a higher percentage of seizure free patients with less complications for those in eloquent brain areas. Nearly a quarter (6/26) of the patients included in our series would have had incomplete resections without having performed iopMRIs. It is of note that four out of these six patients had excellent post-operative seizure control at the time of last follow-up, underlining the benefit of this technique.

Sun and co-workers seem to confirm our hypothesis, especially regarding the fact that fewer complications occur by using iopMRI combined with neuronavigation.^16^ However, only a small percentage of their patients had preoperative epileptic seizures and few suffered from drug resistant epilepsy. Additionally, with an average of 12·6 months, the follow-up period of their study was a quarter of our patients, although seizure outcome of their patients was excellent, too.

### Surgical strategies

Patients with single CMs, who had sporadic, non drug resistant seizures of short duration and low frequency, are complete seizure free with pure lesionectomy in about 60–100%. In patients with chronic epilepsy, seizure-freedom rates after 2 years of follow-up drop to 62·5–68·7% with pure lesionectomy.2,^31^ A retrospective analysis of 163 patients that underwent lesionectomy alone showed a complete seizure-freedom rate of 68·7% after a mean follow-up of 48 months.^28^ If the hemosiderin-stained tissue is also removed, these seizure free rates reach 53–78%.9,11,^12^ However, a current meta-analysis by Englot and colleagues found no statistical difference between the rate of seizure freedom in lesionectomy alone (75%) and extended lesionectomy with additional excision of the hemosiderin rim (76%), without any data from studies including intraoperative imaging.^8^

In the same meta-analysis, drug resistance, secondary generalized seizures and a CM diameter above 1·5 cm was associated with a lower rate of epilepsy freedom (Engel Class I). After a mean follow-up of > 12–97 months, seizure freedom in all patients was 29% above compared to 86% below 1·5 cm. As larger CMs are more difficult to be resected completely, our findings indicate that high field iopMRI leads to a maximal resection and thus to a higher percentage of seizure free patients. Comparing our study with other series performing extended lesionectomies, we report on an excellent long-time seizure control after a mean follow-up of nearly 4 years with 80·8% of all patients being seizure-free. Moreover, we achieved those results although 65% of the patients had secondary generalized seizures, 69% drug resistant epilepsy and 38% a cavernous malformation with a diameter above 1·5 cm.

From the epileptological point of view, seizures in patients harbouring CMs must be carefully classified and the site of the CM and the presence or absence of dual pathology has to be accurately determined to escalate postoperative seizure free rates, as described earlier.^13^ In two of our patients, extended resections had to be performed as a consequence of preoperative epilepsy work-up, and both of them are seizure free to date.

Concerning the postoperative medication status, Komotar and colleagues argued that one of the major concerns about the report on ‘seizure-freedom’ in patients after surgery was the continuation of AEDs in the follow-up period.^31^ Here, our study adds long-term results with respect to the use of AEDs in patients after surgery. Withdrawal of AEDs was possible in 57·1% of our patients with Engel 1A outcome, where the remaining patients of this group took only 1 AED. On the contrary, over three-quarter of all patients reported in the literature are still taking AEDs after 3 years despite seizure-freedom.12,^32^

### Postoperative morbidity

Recent publications report up to 20·6% of patients developing neurological symptoms immediately after surgery.3,9,28,^33^ Fortunately, at follow-up the rate of severe permanent neurological deficits descends to 2·6%–8%.3,4,9,10,12,15,28,32,^33^ Our strategy employing FN and iopMRI only led to two *mild* (7·7%) and one *moderate* (3·8%) permanent visual field deficit, while the rate of post-surgical general complications was 7·7% and *severe* neurological deficits did not occur at all.

### Limitations of the study

This study lacks randomization in both, the surgical technique using FN and iopMRI compared to unguided surgery as well as in comparing pure lesionectomy to additional resection of the hemosiderin rim. However, reports from current literature indicate that the resection of the lesion and the hemosiderin rim are more beneficial for long-term seizure control compared to lesionectomy alone.11,12,^33^ Complete removal of the CM including the hemosiderin rim can be a major problem without neuronavigation and intraoperative resection control by MRI, especially in deep seated lesions near eloquent brain areas. Ultrasound might be an alternative to iopMRI, but in our experience, hemosiderin tissue might not be detectable at that extend compared to high field MR imaging. In the future, prospective randomized controlled trials with larger patient numbers are needed to further clarify the possible benefit of our multimodal intraoperative navigation approach in patients with cerebral cavernoma.

Additionally, usage of FN might contain some inaccuracies, including the inconvenience generated by brain shift.^34^ Shifting of brain structures through loss of cerebrospinal fluid or removal of a large lesion was avoided by updating neuronavigation data by iopMRI in our study. Therefore, intraoperative imaging in our opinion leads to a compensation of these sources of error. Strategies to overcome the negative impact of brain shift on pre- and intraoperative functional imaging data were reported in previous publications of our department.24,^35^ Lastly, we did not choose to perform intraoperative electrocorticography (ECoG) recordings in cases with temporally located cavernomas, which could have raised the good seizure outcome rates of our series even further.^36^

## Conclusion

The key findings of our study are: (1) application of high field 1·5T MRI successfully identifies remnant cavernoma mass and its hemosiderin rim and leads to a supramaximal resection of CMs (2) extended lesionectomy with complete removal of the hemosiderin rim resulted in excellent seizure control and (3) using FN, we achieved an acceptable rate of neurological complications despite the lesions’ vicinity to eloquent brain areas.

## Disclosures

Hajo Hamer has served on the scientific advisory board of Eisai, Pfizer, and UCB Pharma. He served on the speakers’ bureau of Desitin, Eisai, GlaxoSmithKline, and UCB Pharma and received research funding from Desitin, Janssen-Cilag GlaxoSmithKline, and UCB Pharma. Dr. Blumcke has received financial support from the EuroCore EpiGENet consortium (DFG Bl 421/3-1), and received speaker's fee from UCB and Desitin Pharma during the last 24 months. The remaining authors have no conflicts of interest to disclose. The authors do not have any personal financial or institutional interest in any of the drugs, materials, or devices described in this article.
